# Successful repair using thymus pedicle flap for tracheoesophageal fistula: a case report

**DOI:** 10.1186/s40792-018-0458-8

**Published:** 2018-05-23

**Authors:** Yoji Fukumoto, Tomoyuki Matsunaga, Yuji Shishido, Masataka Amisaki, Yusuke Kono, Yuki Murakami, Hirohiko Kuroda, Tomohiro Osaki, Teruhisa Sakamoto, Soichiro Honjo, Keigo Ashida, Hiroaki Saito, Yoshiyuki Fujiwara

**Affiliations:** Division of Surgical Oncology, Department of Surgery, Tottori University Faculty of Medicine, 36-1 Nishicho, Yonago, Tottori 683-8504 Japan

**Keywords:** Thymus pedicle flap, Tracheoesophageal fistula, Esophageal cancer, Post-operative complication

## Abstract

**Background:**

Treatment for tracheoesophageal fistula (TEF), a life-threatening complication after esophagectomy, is challenging.

**Case presentation:**

A 75-year-old man with thoracic esophageal cancer underwent subtotal esophagectomy and gastric tube reconstruction through the post-mediastinal root after neoadjuvant chemotherapy. Owing to postoperative anastomotic leakage, an abscess formed at the anastomotic region. Sustained inflammation from the abscess caused refractory TEF between the esophagogastric anastomotic site and membrane of the trachea, and several conservative therapies for TEF failed. Hence, the patient underwent surgery including division of the fistula, direct suturing of the leakage sites, and reinforcement with the flap of the thymus pedicle. As a result, the abscess and TEF disappeared after surgery and the patient was immediately administered an oral diet and discharged home 103 days after initial surgery.

**Conclusions:**

Although pedicle flaps for the reinforcement of TEF are usually obtained from muscle or pericardium, these flaps need enough lengths to overcome moving distance. We are the first in the existing literature to have successfully treated TEF with surgical repair using a thymus flap located close to TEF. The thymus pedicle might be another candidate for the reinforcement flap in TEF.

## Background

Esophagectomy, which is generally performed as one of the curative treatments for patients with esophageal cancer, is extremely invasive surgery and associated with frequent severe post-operative complications. One life-threating complication is tracheoesophageal fistula (TEF). Post-operative TEF is very rare, and its incidence is approximately 0.3% [1]; however, it is worth discussing because it may lead to surgery-related death through aspiration pneumonia, respiratory failure, or septic shock [2, 3].

Treatment of TEF is difficult because it has various pathogenic backgrounds, i.e., tracheal inflammation, ischemia, direct surgical injury, or erosion caused by mechanical damage from adjacent materials including esophageal stapling [4, 5]. At present, various types of repair procedures have been reported to treat TEF. For example, the muscular flap or pericardiac flap had been used for reinforcement of defects [1, 6]. However, the optimal management of TEF is still controversial. Here, we report a patient who suffered TEF as a post-operative complication due to anastomosis leakage after esophageal cancer surgery and was successfully repaired with a thymic pedicle flap. This is the first report to use thymus flap for surgical repair of TEF caused after esophageal cancer surgery.

## Case presentation

**Fig. 1:**
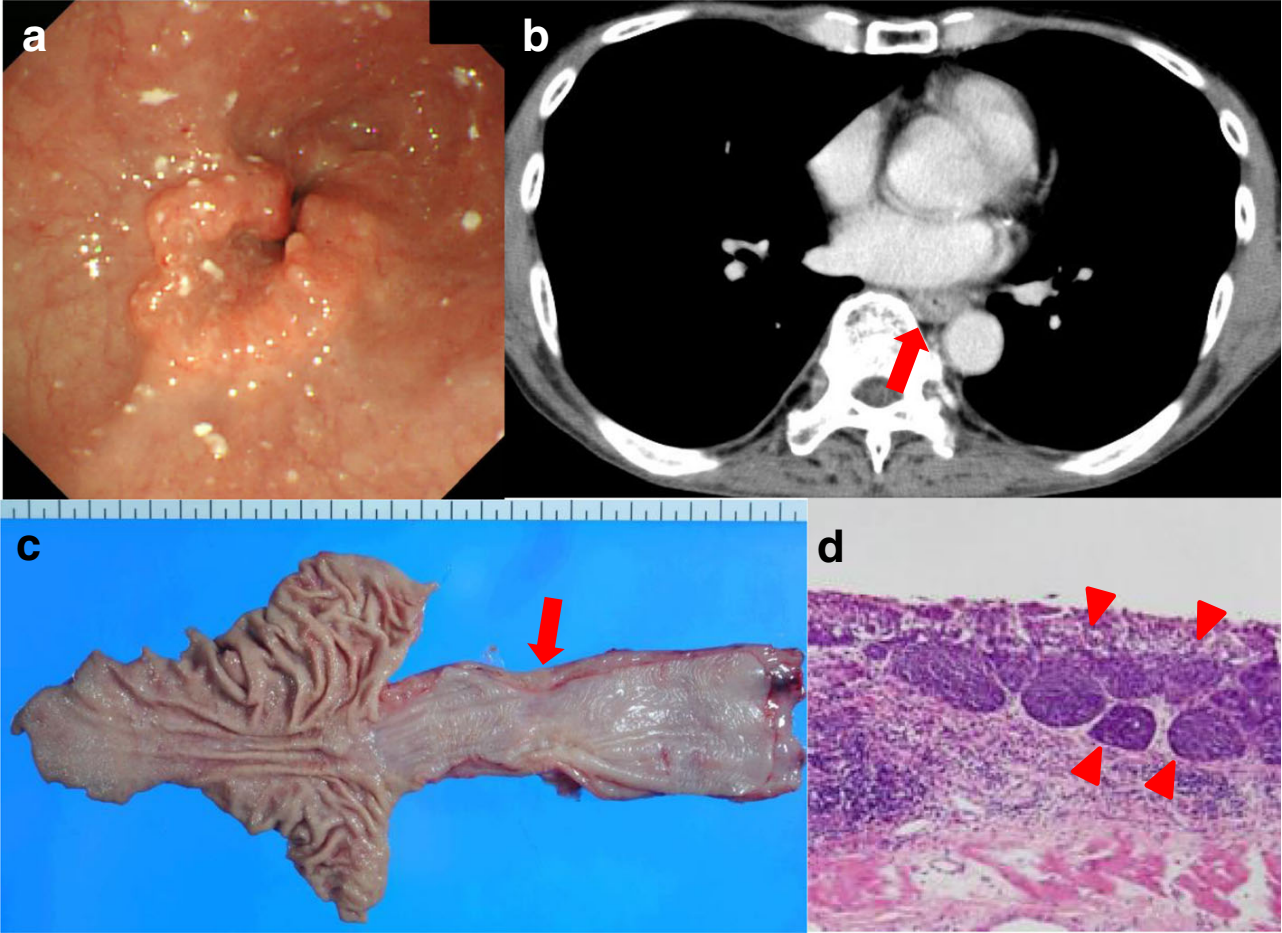
Image findings before intervention and macroscopic and microscopic findings of resected specimen. **a** The endoscopic finding of primary esophageal cancer. Type 3 tumor with esophageal stenosis was located in the lower thoracic esophagus. **b** The preoperative CT image. The arrow indicates neoplastic lesion of the esophagus. **c** The resection specimen. The arrow indicates neoplastic lesion. **d** Hematoxylin-eosin staining of the resected specimen. The arrowheads indicate viable cancer cells within the mucosa of the esophagus

**Fig. 2:**
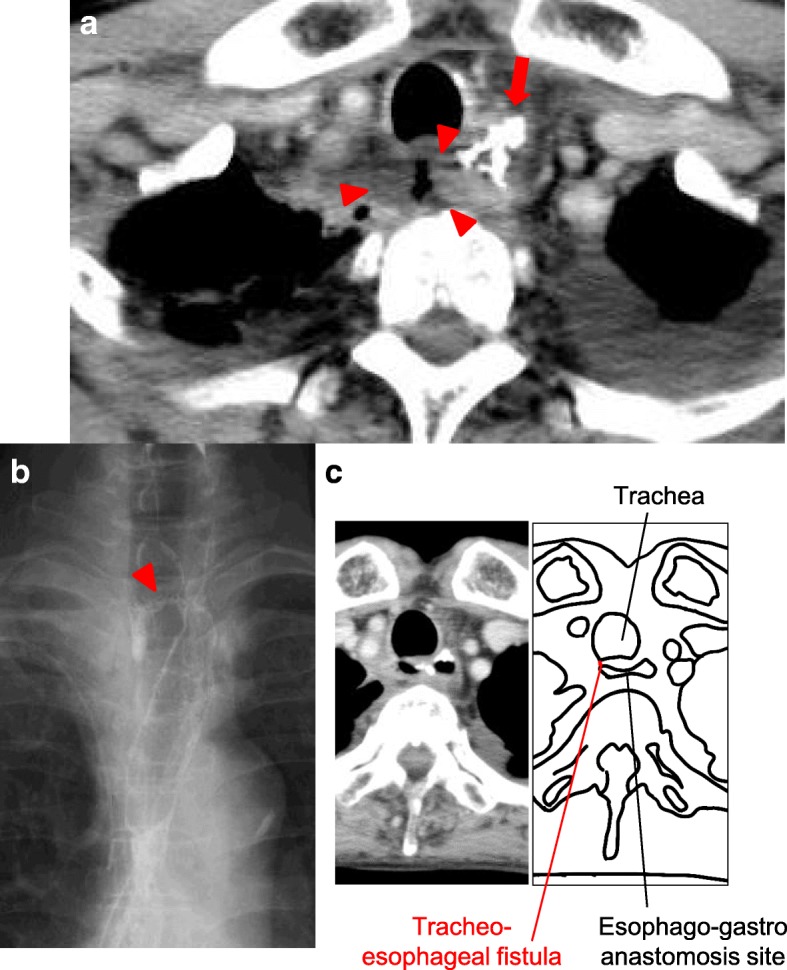
Image findings of postoperative abscess and tracheoesophageal fistula (TEF). **a** The CT shows the leakage of esophagogastric anastomosis. The arrow indicates the hole of leakage and the arrowheads indicate the abscess. **b**, **c** The esophagography shows a TEF. The arrowheads indicate the fistula

**Fig. 3:**
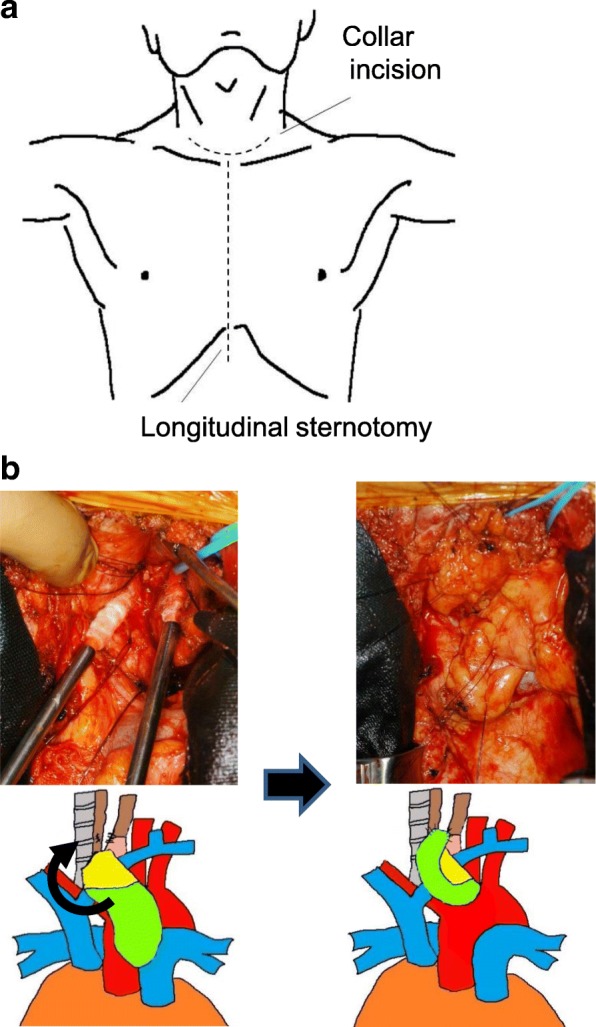
Details of surgery for tracheoesophageal fistula (TEF). **a** The schema of the incision line. **b** The intraoperative findings and schemas. The thymus flap (light green) was sutured on the esophagus (brown)

**Fig. 4:**
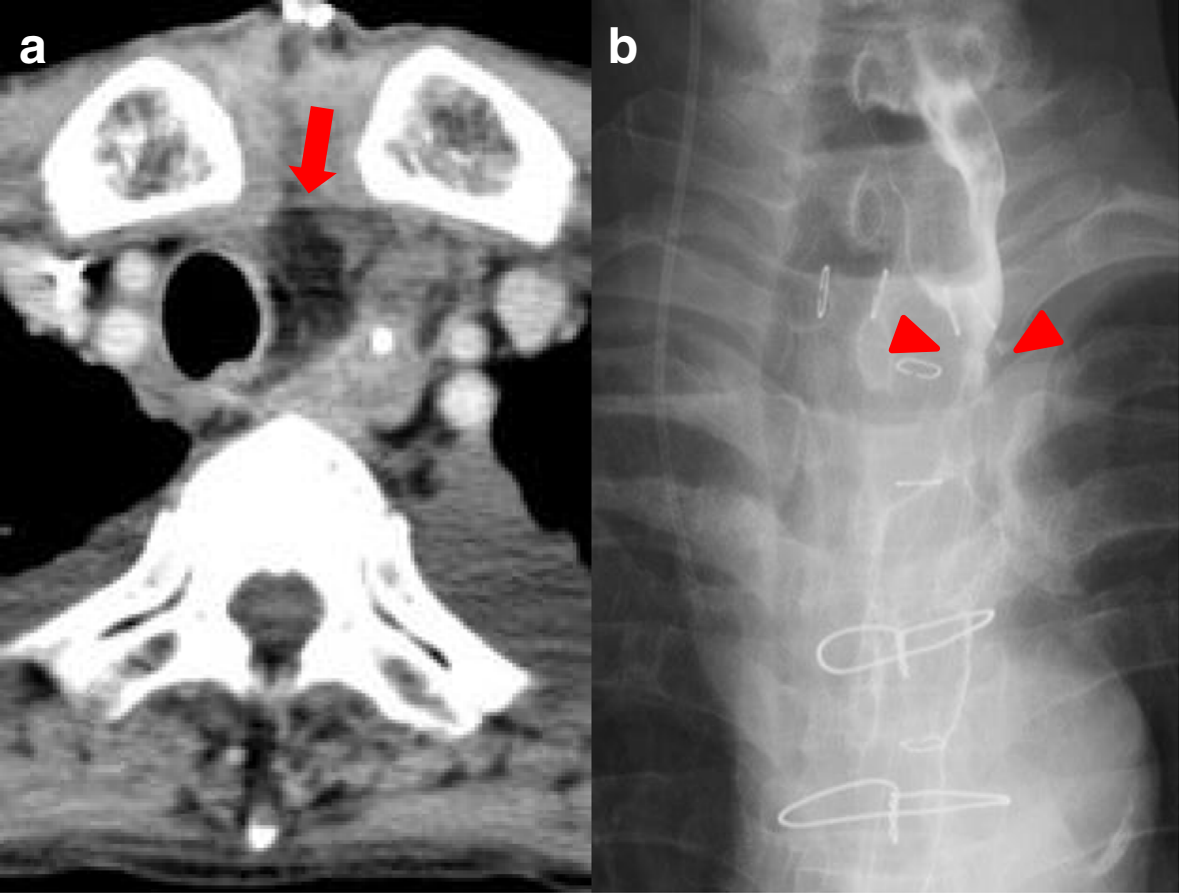
The findings after surgery for tracheoesophageal fistula (TEF). **a** CT image. The arrow indicates the thymus pedicle flap. **b** Esophagography shows no leakage on the esophagogastric anastomotic site (arrowheads)

A 75-year-old man presented with a 5-month history of dysphagia. Endoscopy showed a type 3 tumor with esophageal stenosis at the lower thoracic esophagus (Fig. 1a). Pathological examination with biopsied specimens revealed moderately differentiated squamous cell carcinoma. Computed tomography (CT) showed esophageal wall thickness at lower thoracic esophagus with no lymph node and distant metastases (Fig. 1b). The preoperative diagnosis was clinical T3N0M0 stage II thoracic esophageal cancer [7]. After two courses of preoperative chemotherapy with 5-fluorouracil and cisplatin, the patient underwent subtotal esophagectomy, gastric tube reconstruction through posterior mediastinal route, and three-field lymph node dissection. The esophagogastric anastomosis was achieved using three linear staplers, so-called triangulating stapling technique [8]. The resected specimen is shown in Fig. 1c. A type 3 tumor was located in the lower esophagus, and the pathological examination showed grade 2 pathological effect to neoadjuvant chemotherapy in primary tumor (Fig. 1d) and classified it pathologically as T3N0, pStage II [9]. On post-operative day (POD) 5, elevation of inflammatory reaction and body temperature due to leakage of esophagogastric anastomosis was observed. Esophagography and enhanced CT showed an abscess formation of 2 cm in diameter and a fistula from the posterior anastomotic wall of the esophagus to the abscess (Fig. 2a). Therefore, CT-guided percutaneous drainage from dorsal side of the abscess was performed. On POD 20, fistula from the anastomotic site to main bronchus (TEF) was detected in esophagography (Fig. 2b, c). Fortunately, the patient was asymptomatic except for fever and was conservatively managed with no oral-intake, intravenous total hyperalimentation, and administration of antibiotics. Moreover, octreotide and daily injection of human plasma-derived dried blood coagulation factor XIII were administrated to encourage wound healing. However, TEF continued and on POD 56, the inside of the abscess was endoscopically filled by a coil (0.6 × 20 cm Interlocking Detachable Coil, *Boston Scientific Corp.*), and the TEF was filled with a fibrin glue injection. These procedures achieved temporary disappearance of the TEF. However, the coil was spilling out after a few days, and the closure of TEF was imperfect. Thus, surgical intervention was considered for curative treatment. On POD 70, surgery for the TEF was performed. Under general anesthesia, thoracotomy with longitudinal sternotomy and collar incision of the neck were performed (Fig. 3a). After exposure of the abscess cavity, we detected the right posterior wall of the anastomotic site with the fistula connected to the tracheal membrane of the main bronchi. First, the hole of the anastomotic leakage site and the tracheal membrane were sutured with 4-0 monofilament absorption thread. We noticed that the thymus pedicle had enough volume, of which the maximum thickness in preoperative CT was 1.8 cm, and was located closer to the leakage sites than the pectoralis major muscle. Then, a flap made by mobilizing the left lobe of the thymus, but not its feeding vessels, was fixed to repair sites of anastomotic leakage without visually impairment blood flow and the tracheal membrane using 4-0 monofilament absorption threads to reinforce the repair sites (Fig. 3b). After surgery, the abscess cavity had disappeared and TEF had healed (Fig. 4a, b, POD 77). The patient then started an oral diet and was discharged home on 103 POD.

### Discussion

We report a patient who developed a fistula between the esophagogastric anastomosis and the membranous portion of the bronchi after esophagectomy for esophageal cancer. In this case, we had tried conservative treatments for TEF; however, they were unsuccessful. Then, we used a surgical approach and were able to treat the TEF by using fistulectomy, a running suture of fistula holes, and reinforcement of the bronchi with a pedicle flap of the thymus.

Conservative therapy is the first option for TEF with mild symptoms. A less invasive treatment such as fibrin glue has a potential to be a successful treatment of refractory fistula [7]. There have been some reports that the tracheal fistula could be closed with both fibrin glue and tracheal stent insertion as a conservative treatment. Additionally, in this case, the abscess that was the main cause of TEF was first drained, followed by administration of fibrin glue. However, these procedures failed to cure TEF. The main problem of conservative therapy for TEF is that the conservative method might be defeated by positive mechanical pressure from the respiratory tract or esophagogastric tract. Therefore, surgeons should consider surgical treatment for TEF. According to the literature, if the fistula fails to be cured within 4–6 weeks, patients should be treated surgically [10].

The tissue flap is widely used in the field of other than gastrointestinal surgery [1, 6, 11]. Indeed, the advantage of a tissue patch such as the muscular flap for head and neck reconstruction is well known [1, 6] because such vascularized tissue can be easily mobilized and adhere to other tissues. There have also been reports that the thymus flap is useful for covering tissue of the bronchial stump in tracheal reconstructive surgery [10]. The thymus flap is located close to the bronchi and has good vascular flow and sufficient volume compared with other tissues such as muscle flaps such as pleural flap, diaphragmatic flap, and azygos flap. However, there are some disadvantages to use as the thymus flap. The thymic lobes in adulthood sometimes present an atrophied fibrofatty tissue and less volume for patch reinforcement. The branches of the mammary arteries supplying blood flow to the thymus restrict thymus flap mobility. Furthermore, the flap length of the thymus is sometimes not long enough [12]. Therefore, there have been few reports of the thymus flap being used for the reconstructive tissue patch for TEF in past literature. In this case, the thymus could be used as a covering tissue patch for TEF reinforcement. The reason was that we performed longitudinal sternotomy to reach a TEF located at the cervicothoracic boundary and deep in the mediastinum. The sternotomy, rather than thoracotomy, divided loose adhesion of peri-thymic tissue and improved mobilization of the thymus. The most important reason was the condition of the thymus in the present case. The thymus in this case had the maximum thickness of 1.8 cm in preoperative CT, which was greater than the maximum thickness of 1.3 cm as a mean value of an adult male at the same age [13], and the nearest part of thymus to TEF had enough strength with mean thickness of 0.8 cm in CT. Moreover, the thymus was anatomically shifted toward the right side, which allowed it to move and to cover the TEF site. These factors might contribute to the successful treatment of TEF with thymus flap.

## Conclusions

Management of a TEF requires great knowledge and skill. In this case, a life-threating complication was successfully treated with surgical repair using a thymus flap. In conclusion, the thymus flap could be a promising option for reconstruction and reinforcement of a TEF after esophagectomy, if preoperative CT shows enough volume of thymus tissue for reinforcement.

## Abbreviations

CT: Computed tomography
POD: Post-operative day
TEF: Tracheoesophageal fistula
